# Spiral sound-diffusing metasurfaces based on holographic vortices

**DOI:** 10.1038/s41598-021-89487-8

**Published:** 2021-05-13

**Authors:** Noé Jiménez, Jean-Philippe Groby, Vicent Romero-García

**Affiliations:** 1 Instituto de Instrumentación para Imagen Molecular, Universitat Politècnica de València, Consejo Superior de Invesigaciones Científicas, Camino de Vera S/N, 46085 Valencia, Spain; 2 Laboratoire d’Acoustique de l’Université du Mans (LAUM), UMR CNRS 6613, Institut d’Acoustique-Graduate School (IA-GS), CNRS, Le Mans Université, Le Mans, France

**Keywords:** Applied physics, Metamaterials

## Abstract

In this work, we show that scattered acoustic vortices generated by metasurfaces with chiral symmetry present broadband unusual properties in the far-field. These metasurfaces are designed to encode the holographic field of an acoustical vortex, resulting in structures with spiral geometry. In the near field, phase dislocations with tuned topological charge emerge when the scattered waves interference destructively along the axis of the spiral metasurface. In the far field, metasurfaces based on holographic vortices inhibit specular reflections because all scattered waves also interfere destructively in the normal direction. In addition, the scattering function in the far field is unusually uniform because the reflected waves diverge spherically from the holographic focal point. In this way, by triggering vorticity, energy can be evenly reflected in all directions except to the normal. As a consequence, the designed metasurface presents a mean correlation-scattering coefficient of 0.99 (0.98 in experiments) and a mean normalized diffusion coefficient of 0.73 (0.76 in experiments) over a 4 octave frequency band. The singular features of the resulting metasurfaces with chiral geometry allow the simultaneous generation of broadband, diffuse and non-specular scattering. These three exceptional features make spiral metasurfaces extraordinary candidates for controlling acoustic scattering and generating diffuse sound reflections in several applications and branches of wave physics as underwater acoustics, biomedical ultrasound, particle manipulation devices or room acoustics.

## Introduction

The control of the acoustic scattering is at the origin of a wide range of practical applications, from architectural to underwater acoustics. In the last years, locally-reacting flat-surfaces composed of subwavelength resonators, i.e., metasurfaces, have been actively developed and offer a wide range of possibilities for manipulating reflected wavefronts^1–3^. Metasurfaces allow the simultaneous control of the phase and amplitude of the reflected field^4^. Negative refracting metasurfaces^5^, scattering-free refractive devices^6^, non-specular reflecting surfaces^7^, subwavelength focusing^8^, beamforming devices^9,10^, cloaking^11^ or broadband and perfect sound absorbers using subwavelength panels^12–15^ have been reported.

Nowadays, research on acoustic metasurfaces is very active. However, the use of locally resonant structures to control sound diffusion in room acoustics dates back to the late 70’s, when arrangements of quarter-wavelength resonators, called phase-grating diffusers, were introduced by Schröeder to generate diffuse reflections^16^. These acoustic devices have found practical applications in room acoustics and are widely used in many broadcast studios, modern auditoria, music recording, control, and rehearsal rooms^17^. The scattering pattern of a panel is essentially driven in the far field by the Fourier transform of its spatially-dependent reflection coefficient. In this way, reflecting screens based on number theory sequences with flat spatial Fourier transform were proposed to generate diffuse reflections. These sequences can be bipolar, binary^18^, ternary and quadriphase^19^ or quadratic-residue types^17^. Sequences also exist, whose first component of the spatial Fourier transform is equal to zero. These sequences are of interest because the specular reflection vanishes in this situation, as it does in primitive root or index sequence diffusers^17^. However, the performance of these traditional non-specular sound diffusers is limited because this effect only occurs at the design frequency and multiples of it, with exception to critical frequencies. Recently, metamaterials were proposed to reduce the thickness of Schröeder diffusers by using Helmholtz resonators instead of quarter-wavelength resonators^20^ or slow-sound metasurfaces with deep-subwavelength resonators^21,22^.

In this work, we study the scattering properties of spiral metasurfaces based on holographic acoustic vortices and make use of them to design broadband and non-specular sound diffusing surfaces. Acoustic vortices are wave fields containing phase singularities^23^, the rotation phase of which is $\exp (il\phi )$, with $\phi$ the azimuthal angle and *l* the topological charge of the vortex. Vortex beams have found applications in the rotation of objects^24–28^, the trapping and manipulation of particles^29–33^ or in acoustic communication systems for transmitting coded information^34^. Several approaches have been proposed to generate acoustic vortex beams, including active sources^35,36^ or passive structures such as helicoidal surfaces^35,37^, locally-resonant metamaterials^38–41^, acoustic delay lines^42^, or acoustic holograms^43–45^. Vortices can also be generated using Archimedean spiral gratings^46–48^, or Fresnel spiral gratings to produce sharply focused vortex beams^49^. However, the acoustic scattering by spiral structures has not been explored previously.

Vortex beams present a null in the far field because the phase singularity of a vortex beam inhibits the propagation of waves along the axial direction. In this way, reflecting surfaces based on vortices only present off-axis reflections. In addition, these spiral metasurfaces can spread uniformly the energy over the entire angular spectrum by focusing (or defocusing) a vortex in the near field, thus, allowing the design of ultra-broadband acoustic diffusers with simultaneous high diffusion performance and non-specular reflections.

**Figure 1:**
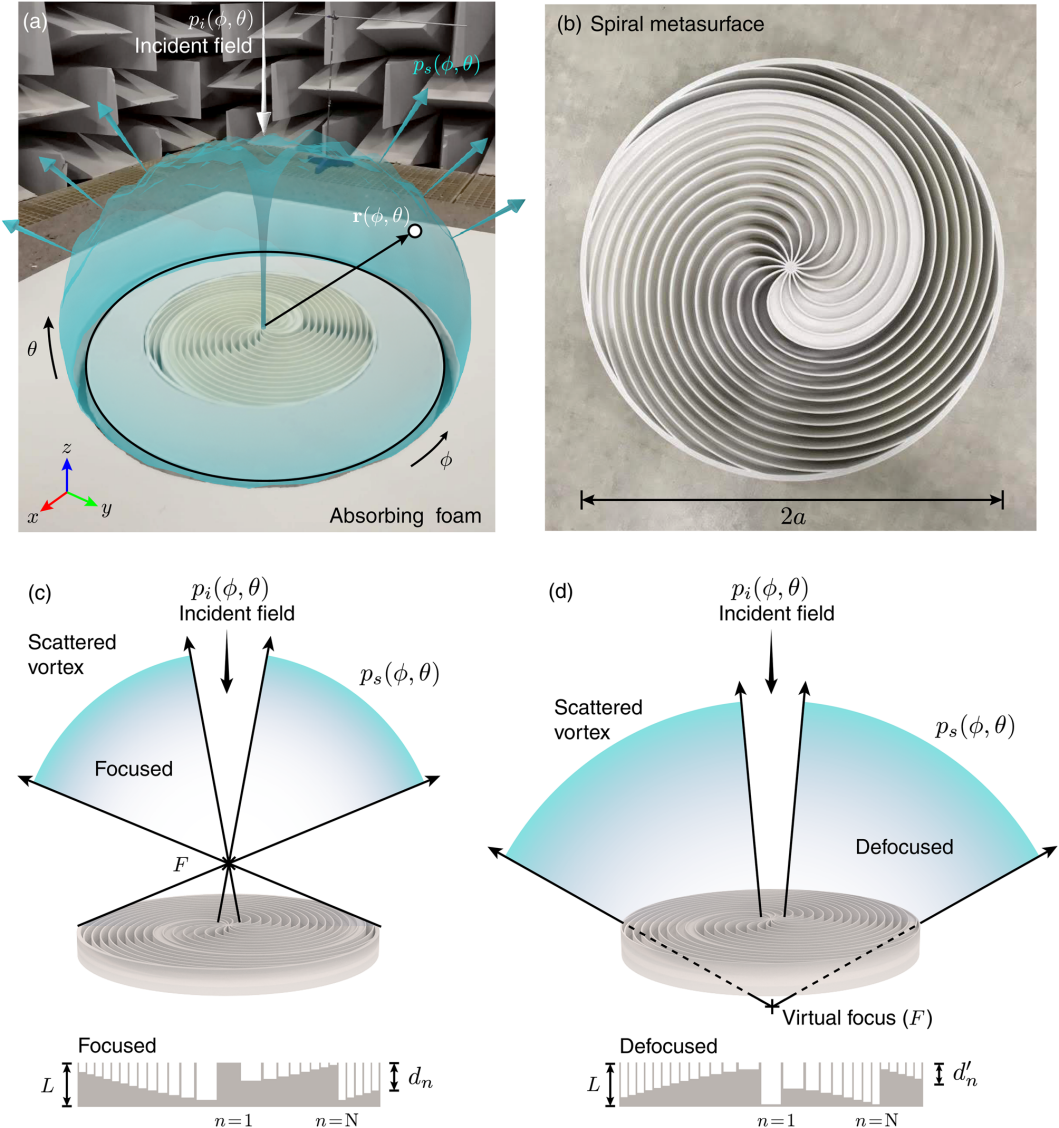
(**a**) Scheme of the proposed spiral-shaped sound diffusing metasurface. (**b**) Geometry of the panel for the focusing configuration. (**c**) Spiral metasurface for the focusing configuration and its geometry. (**d**) Spiral metasurface designed for the defocusing configuration using a virtual image of a vortex and its geometry.

## Results

### Holographic-vortex metasurfaces

The proposed metasurface is sketched in Fig. 1a,b. The structure consists of a circular flat panel of radius *a* and thickness *L* and has *N* wells of spiral shape. Each well is indexed by $n=1,2,3,\ldots ,N$ and is of constant depth $d_n$. We propose two different structures: one that focuses a vortex in the near field on top of it, as shown in Fig. 1c, and another that virtually focuses a vortex behind it, as shown in Fig. 1d.

The field pattern generated by a spherically focused vortex source located at a distance $z=F$ on the metasurface plane $z=0$ can be approximated in cylindrical coordinates $\mathbf{r}=\mathbf{r}(\phi ,r,z)$ by a hyperbolic phase profile as^50^

1 $$\begin{aligned} p(\phi ,r) = \frac{-ip_0}{k\sqrt{r^2 + F^2}}\exp \left( i k\sqrt{r^2 + F^2}\right) \exp ( il\phi ), \end{aligned}$$

where *F* is the focal distance, $k = \omega /c_0$ is the wavenumber, $\omega$ is the angular frequency, $c_0$ is the sound speed, and $p_0$ is a constant. The time convention in this work is $\exp (-i\omega t)$. If a surface is set to radiate a time-reversed (or complex conjugate in the frequency domain) version of this field, a diffraction-limited vortex converging at the focal point $z=F$ will be observed, because of the time-invariance of the acoustic equations. On the one hand, in the case of a real focal point when $F>0$ a diffraction-limited focused vortex beam is generated, as sketched in Fig. 1c. On the other hand, when $F<0$ the resulting field diverges spherically from the metasurface and defocusing is observed as shown in Fig. 1d with a virtual focal point. Note that no phase-conjugation is needed in Eq. (1) in the defocusing case, because the holographic field already captured the diverging wavefront. Therefore, defocusing should present inverse phase curvature and inverse topological charge.

In order to design a metasurface with such phase profile, we follow a two step procedure. The first step consists in spatially discretizing the metasurface with a geometry compatible with the phase profile of Eq. (1). This can be done by the expansion of the binary Fresnel-spiral zone plates^49^ for the case of the *N* phase zones and $l_0$ arms. The boundary between the $n-1$ and *n*-th phase zone is then given by the following expression

2 $$\begin{aligned} r_{n,m}(\phi ) = \sqrt{\left[ F + \lambda _0 \left( \frac{ l_0 \phi }{2\pi } + \frac{n }{N} + m \right) \right] ^2 - F^2}, \end{aligned}$$

where $n=0,\ldots ,N-1$ is the index of each wall, $0<\phi <2\pi$ is the azimuthal coordinate, $\lambda _0 = c_0/f_0$ is the design wavelength with $f_0$ the design frequency, $l_0$ represents the topological charge at the design frequency, and $m=0,1,\ldots ,l_0-1$ is the index of each arm.

**Figure 2:**
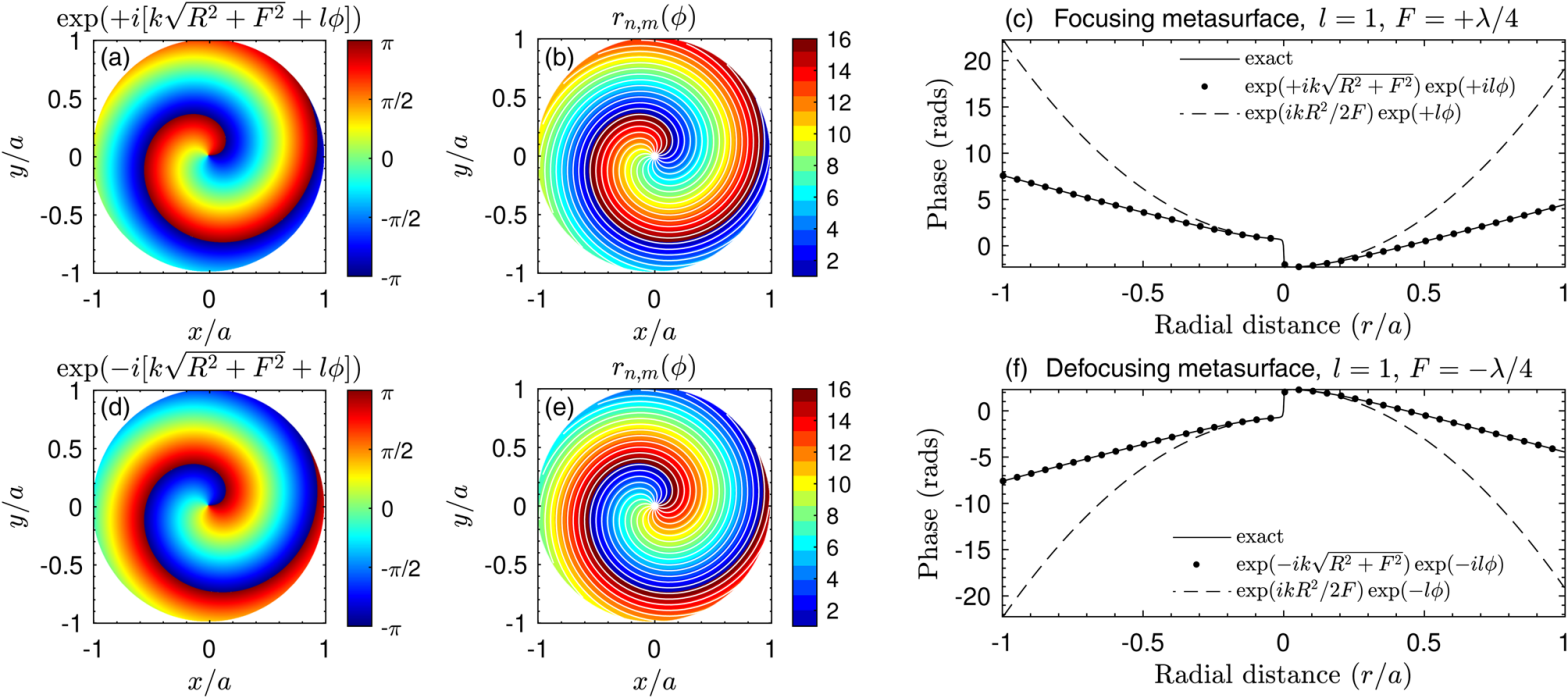
(**a**) Scheme of the proposed spiral-shaped sound diffusing metasurface. (**b**) Geometry of the panel for the focusing configuration. (**c**) Spiral metasurface for the focusing configuration and its geometry. (**d**) Spiral metasurface designed for the defocusing configuration using a virtual image of a vortex and its geometry.

On the one hand, the focusing metasurface synthesizes a wavefront converging towards the focal point. Figure 2a shows the phase given by Eq. (1), while Fig. 2b shows its corresponding phase zones for, e.g., $N=16$ zones. The white lines in Fig. 2b correspond to the polar curves given by Eq. (2). It can be observed that each polar curve fits to the boundary between adjacent phase zones. Indeed, Fresnel-spirals^49^ are the exact zone plates for focused vortices. In Fig. 2c the hyperbolic phase distribution given by Eq. (1) is compared with the exact phase-conjugated projection of a monopole source located at the focus. Note that both phase distributions agree, while a parabolic approximation fails to describe the exact focusing phase for highly focused metasurfaces. On the other hand, the phase distribution for a defocused metasurface is shown in Fig. 2d. Because this metasurface should synthesize a field diverging away from the virtual focus, phase conjugation is applied, resulting in the inversion of the phase and the topological charge, as shown also in Fig. 2e for the phase zones. Note that the curvature of the phase profile along the radial coordinate for the defocusing metasurface is inverted as compared with the focusing case, see Fig. 2f.

The second step consists in assigning to each phase zone the phase values given by Eq. (1). In fact, the focusing metasurface corresponds to the case with exp($il\phi$), while for the defocusing case to exp($-il\phi$). In this work, each phase zone is made of a quarter-wavelength resonator, the thickness of which is limited by the rigid walls located at $r_n$ (Eq. 2). Therefore, the metasurface is made of spiral-shaped wells acting as quarter-wavelength resonators. The control of the value of the phase in each well can be fixed by its depth, $d_n$. In fact, the reflection coefficient at normal incidence of each well in the metasurface is given by $R_n = (Z_0 -i{\bar{Z}}_n\cot k_n d_n )/(Z_0 + i{\bar{Z}}_n\cot k_n d_n)$, where $k_n$ and ${\bar{Z}}_n$ are respectively the complex and frequency dependent wavenumber and the acoustic impedance of the *n*-th well accounting for the viscothermal losses^51^, and $Z_0$ is the impedance of the surrounding medium (see more details in section “Methods”). The depths $d_n$ and $d_n'$ of the *n*-th well, for the focusing and defocusing panels respectively, are set accordingly to

3 $$\begin{aligned} d_n = \frac{n\lambda _0}{2N} \quad \mathrm {and}\quad d_n' = \frac{(N-n+1)\lambda _0}{2N}, \end{aligned}$$

to produce a reflection coefficient whose phase follows the distribution given by Eq. (1). In Eq. (3), the design wavelength $\lambda _0 = 2L$ is associated to the lowest cut-off frequency of the structure and $f_0 = c_n/2L$, where $c_n$ is the sound speed inside the well.

The Rayleigh–Sommerfeld equation and the Fourier–Fraunhofer approximation (see more details in section “Methods”) are used to theoretically evaluate the scattered field in the near field and in the far field, respectively. The structure is thus designed by fixing the lowest working frequency, $(f_0)$ the focal distance (*F*), the number of slits (*N*), the panel radius (*a*) and the topological charge at the design frequency ($l_0$). In this work, spiral metasurfaces composed of $N=16$ wells, with $l_0=1$, $f_0 = 2$ kHz, $|F| = \lambda _0/4 = 4.3$ cm, and $a=22.5$ cm are designed to be efficient in air. The total thickness of the structure is thus $L = 8.5$ cm.

### Near-field vortex focusing and defocusing

**Figure 3:**
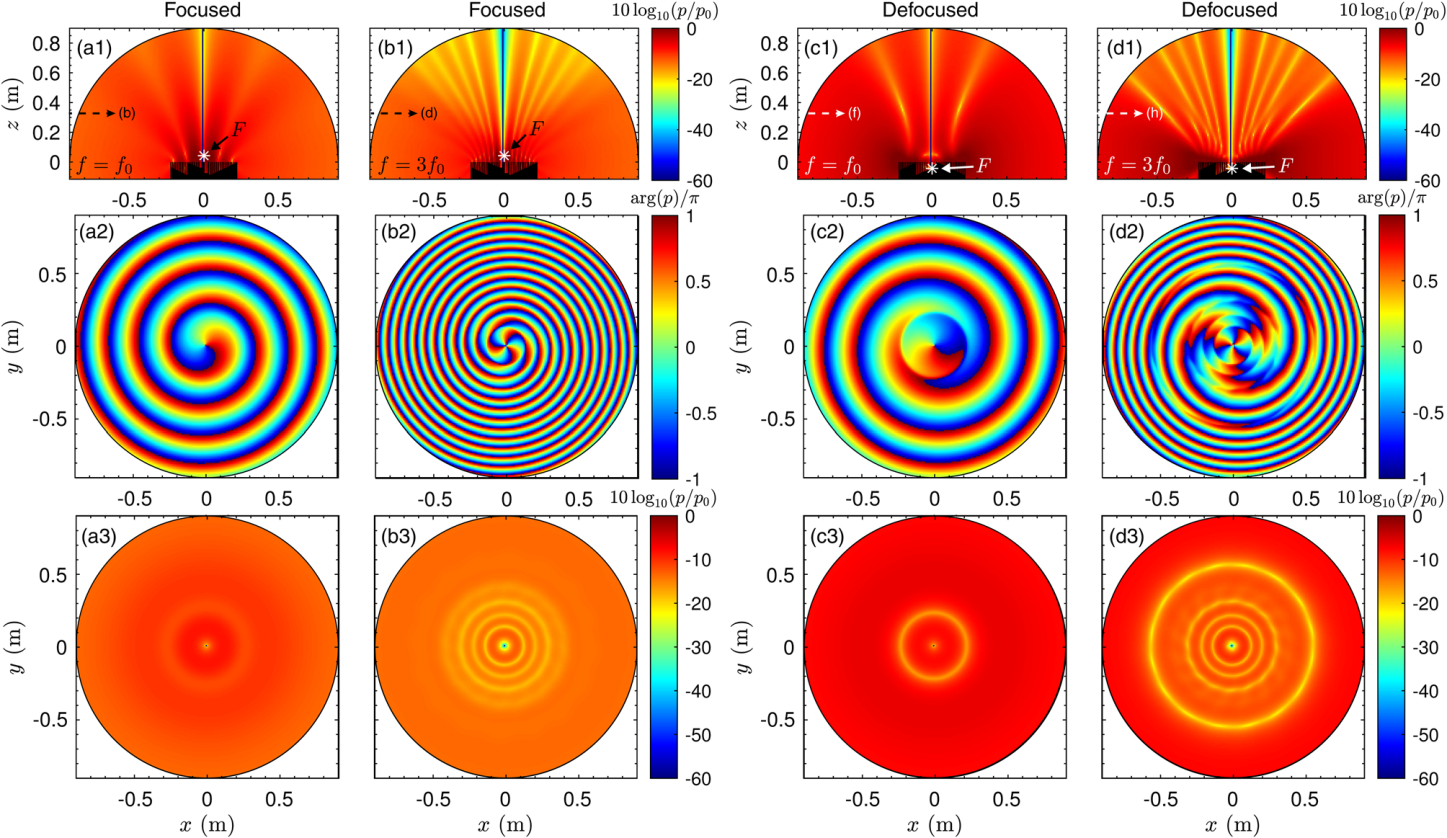
(**a1**) Scattered field for the focusing metasurface in the near field at the design frequency $f=f_0$. (**a2**) Phase and (**a3**) magnitude of the scattered field in the transversal plane $z=2a$. (**b1**) Scattered field for the focusing metasurface in the near field at frequency $f=3f_0$. (**b2**) Phase and (b3) magnitude of the scattered field in the transversal plane $z=2a$. (**c1**) Scattered field for the defocusing metasurface in the near field at the design frequency $f=f_0$. (**c2**) Corresponding phase and (**c3**) magnitude in the transversal plane $z=2a$. (**d1**) Scattered field for the defocusing metasurface in the near field at frequency $f=3f_0$. (**d2**) Corresponding phase and (**d3**) magnitude in the transversal plane $z=2a$.

We start by analyzing the scattered field in the near field. If a lossless medium is considered, $Z_n=Z_0$, $k_n = k$, the reflection coefficient of the *n*-th well is given by $R_n = \exp (i kd_n )$ and a complete phase change is produced along the *N* wells at the design frequency. For a normal-incidence plane wave the reflection coefficient along the surface matches the holographic field given by Eq. (1). The resulting scattered field at the design frequency (2 kHz) is shown in Fig. 3a1 for the real focusing case ($F>0$). The spiral metasurface generates a scattered field that focuses at the focal spot. The field vanishes at the centre of the structure because of the destructive interference of the scattered waves by the spiral geometry. The phase of the field in the cross-sectional plane at a height of $z=2a$ is shown in Fig. 3a2. A phase dislocation along the axis, which corresponds to a vortex of topological charge $l=1$, is clearly visible. As the focusing spot is very close to the surface (4.3 cm), the wavefront quickly diverges and the magnitude of the field is thus highly uniform after a very short distance, z = 45 cm, as shown in Fig. 3a3, except at the location of the phase dislocation, where the magnitude of the field vanishes due to destructive interferences.

Interestingly, complete phase loops are achieved at frequencies that are integer multiples of the design frequency, $f = mf_0$ along *N*/*m* wells, because the reflection coefficient of quarter-wavelength resonators presents a linear phase. The scattered field at three times the design frequency, i.e., $f=3f_0$ (6 kHz), is shown in Fig. 3b1. It presents a focal spot at $z=F$, and focuses sharply, because diffraction effects are weaker for $f>f_0$. The corresponding scattered field phase in the cross-sectional plane is shown in Fig. 3b2. A phase dislocation is also visible at the centre, but this time the phase performs 3 complete loops along an azimuthal turn, i.e., the topological charge of the scattered vortex is $l = f/f_0$. This relation is fulfilled at multiples of the design frequency for frequencies $0<f/f_0\le N/2$, and $l = f/f_0 - N/2$ for frequencies in the range $N/2>f/f_0>N$. This phenomenon is relevant for spiral metasurfaces because vortices in the normal directio appear periodically in frequency up to $f=Nf_0$. Note that as the topological charge of the scattered vortex increases, so does the hollow area of the field, as shown e.g. in Fig. 3b3. In addition, as quarter-wavelength resonators are impedance matched to the air when the walls between the wells are thin, the structure also scatters vortices at non-integer multiples of the design frequency, see Supplementary material 1 for a deeper analysis. Therefore, these structures present a broadband response.

The scattered field of the virtual defocusing case ($F<0$) is shown in Figs. 3c1–c3. The field near the structure does not focus on a single spot but rather diverges away from the structure. Along the axis of the metasurface, the field also vanishes due to destructive interference and the phase dislocation shows a topological charge of $l=-f/f_0$. Note the topological charge phase has the opposite sign to the focusing case, because it occurs in the holographic field used for the design (Eq. 1).

### Far-field vortex scattering

**Figure 4:**
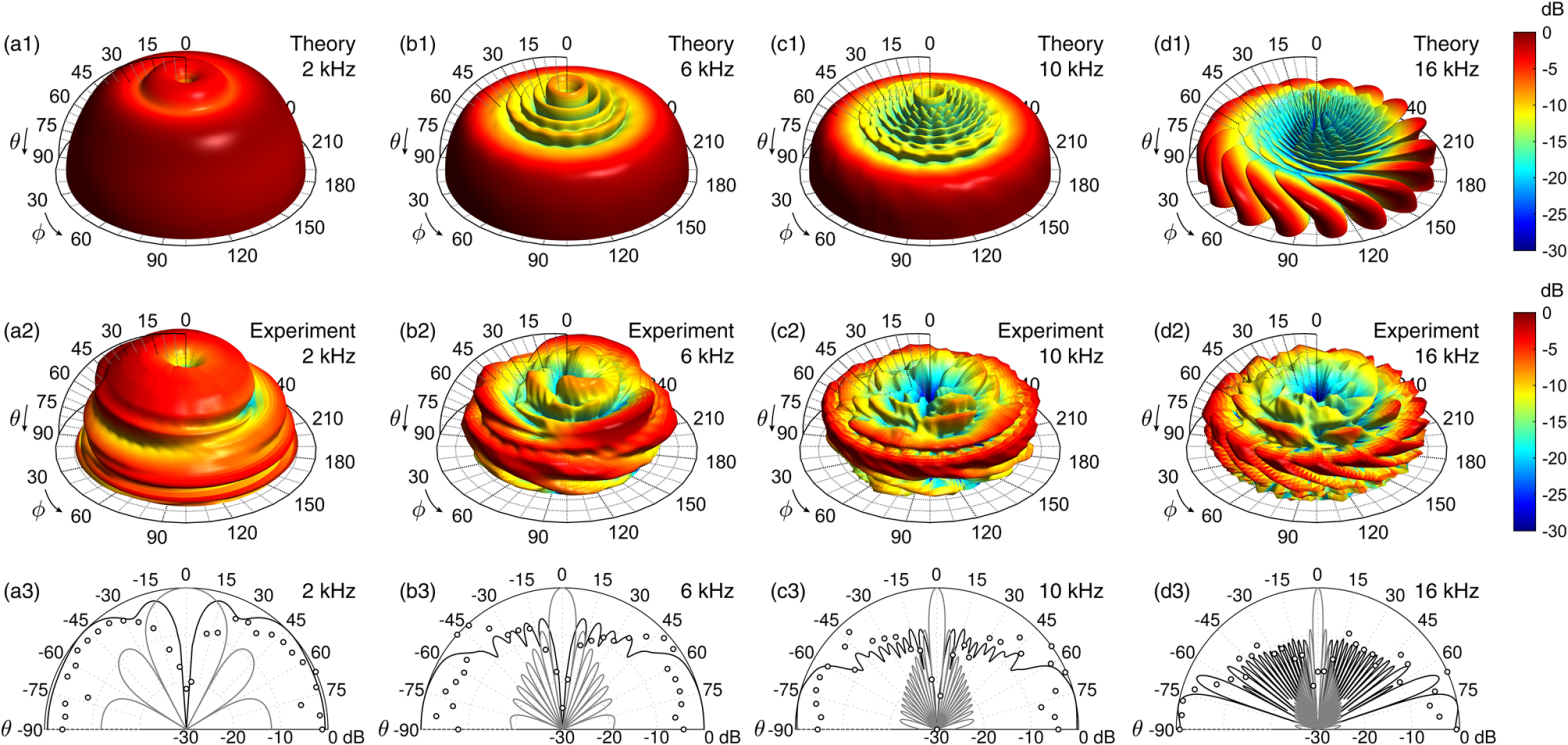
Scattering in the far field at 2 kHz obtained (**a1**) theoretically and (**a2**) experimentally. (**a3**) Polar scattering at 2 kHz obtained experimentally (markers), theoretically (continuous line) and theoretical scattering of a flat panel of same dimensions. (**b1**–**b3**) Corresponding scattering in the near field at 6 kHz, (**c1**–**c3**) at 10 kHz and (**d1**–**d3**) at 16 kHz.

A focusing spiral metasurface was designed ($F = \lambda _0/4$) and manufactured using a selective laser sintering 3D printer. The walls are assumed to be perfectly rigid (see more details in the section “Sample fabrication”). The scattered field by the structure was measured following the ISO-17497 procedure. In the far field, the resulting polar curves are shown in Fig. 4. The scattered field by a circular reflector of the same dimensions is shown for comparison on the polar plots in Fig. 4a3,b3,c3,d3. At the design frequency, Figs. 4(a1-a3), the polar distribution of the scattered waves is uniform when compared to that of the reference reflector, and a reasonable agreement between theoretical and experimental responses is observed. However, all scattered waves interfere destructively in the specular direction ($\theta =0$) in the far field because a vortex is generated in the near field. Therefore, metasurfaces based on holographic vortices inhibit specular reflections because the field presents a phase dislocation in these directions. In addition, the scattering function for all other angles is uniform in the far field, since the waves diverge spherically from the focal point.

The scattered field at frequencies 6 kHz ($l=3$), 10 kHz ($l=5$) and 16 kHz ($l=8$) is depicted in Fig. 4b1–b3,c1–c3,d1–d3, respectively. The amplitude of the scattered field decreases over a wider range of near-normal angles as the frequency increases. This behavior is expected, because vortices of high topological charge present wider nulls, and the range of angles with reduced amplitude is wider in the far field. A time-domain representation of the scattered field using a pulse-burst excitation of frequencies $lf_0$ with $l=1,2,\ldots ,8$ is given in the supplementary videos. The experimental data in the time domain agrees with the theory after inverse Fourier transformation. In each video, it can be identified the scattered field pattern with a vortex of integer topological charge.

**Figure 5:**
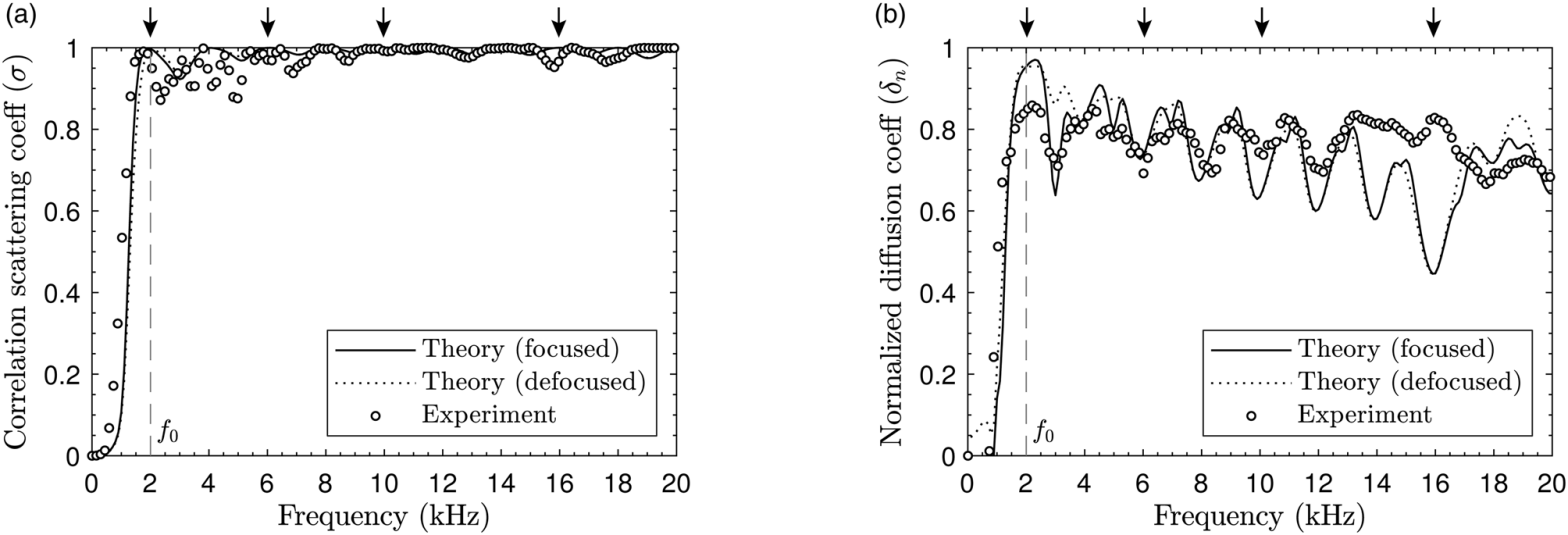
(**a**) Correlation scattering coefficient ($\sigma$) as a function of the frequency. Arrows indicate the frequencies 2 kHz, 6 kHz, 10 kHz, and 16 kHz. (**b**) Normalized diffusion coefficient ($\delta _n$) as a function of the frequency.

### Broadband sound diffusion by holographic vortices

To quantify the performance of the metasurface, the correlation-scattering coefficient, $\sigma (f)$, is calculated as usual in room acoustics and sound diffusers design^17^. This coefficient measures the decorrelation between the scattered field by the structure and that by a flat panel of the same dimensions. Thus, a 0 value of $\sigma (f)$ indicates that the reflection is specular while a 1 value indicates that the scattered energy spreads in all directions other than specular. The retrieved frequency-dependent correlation-scattering coefficient is shown in Fig. 5a. A good agreement is found between theoretical predictions for the focusing and defocusing devices as in the far field both systems present similar scattering field. The experimental results for the focusing device validate this behavior. We observe that the absence of specular reflection makes the correlation-scattering coefficient being almost unitary at frequencies that are multiples of the design frequency because vortices of integer charge are then generated. However, the structure also efficiently scatters vortices at other frequencies (see Supplementary material 1), because it is composed of quarter-wavelength resonators. Therefore, the correlation-scattering coefficient remains close to unity over the entire design frequency band ($\sigma (f)>0.9$ for $f_0<f<Nf_0$). The correlation-scattering coefficient takes a mean value of 0.98 (0.99 in theory) over the frequency range from 2 to 16 kHz.

A second important parameter to quantify the performance of the acoustic structure is the diffusion coefficient, $\delta (f)$, which is widely used in practical applications such as in room acoustics^17^. This coefficient measures the uniformity of the scattering. When all the energy is reflected in a single direction (not necessarily the specular one), $\delta (f)=0$, while $\delta (f)=1$ when there is no preferred direction of reflection and the scattering function is uniform. Note that small panels also generate diffuse reflections due to diffraction by their bounds. The magnitude of the diffusion coefficient is thus normalized by that of a perfect reflector of the same dimensions, namely the normalized diffusion coefficient $\delta _n(f)$. Figure 5b shows the normalized diffusion coefficient calculated theoretically for the focusing and defocusing metasurfaces, and measured experimentally. This coefficient presents a peak at the design frequency of amplitude $\delta (f_0) \approx 0.95$ theoretically and $\delta (f_0) \approx 0.85$ experimentally. This high value arises from the fact that the holographic vortex generates spherically diverging waves. However, the value of the normalized diffusion coefficient cannot reach unity, because there is a lack of scattering in the normal incidence. As the topological charge of the scattered vortex increases with frequency a wider range of angles close to normal direction presents reduced scattering. Therefore, the response is less uniform and the value of the normalized diffusion coefficient decreases with frequency. Note that peaks do not appear at frequencies that are an integer multiple of the design frequency, because at these frequencies the structure scatters multiple vortices at different angles, leading to a uniform scattering pattern. The normalized diffusion coefficient takes a mean value of 0.76 (0.73 in theory) over the frequency range from 2 to 16 kHz.

### Topological charge of the scattered vortices

**Figure 6:**
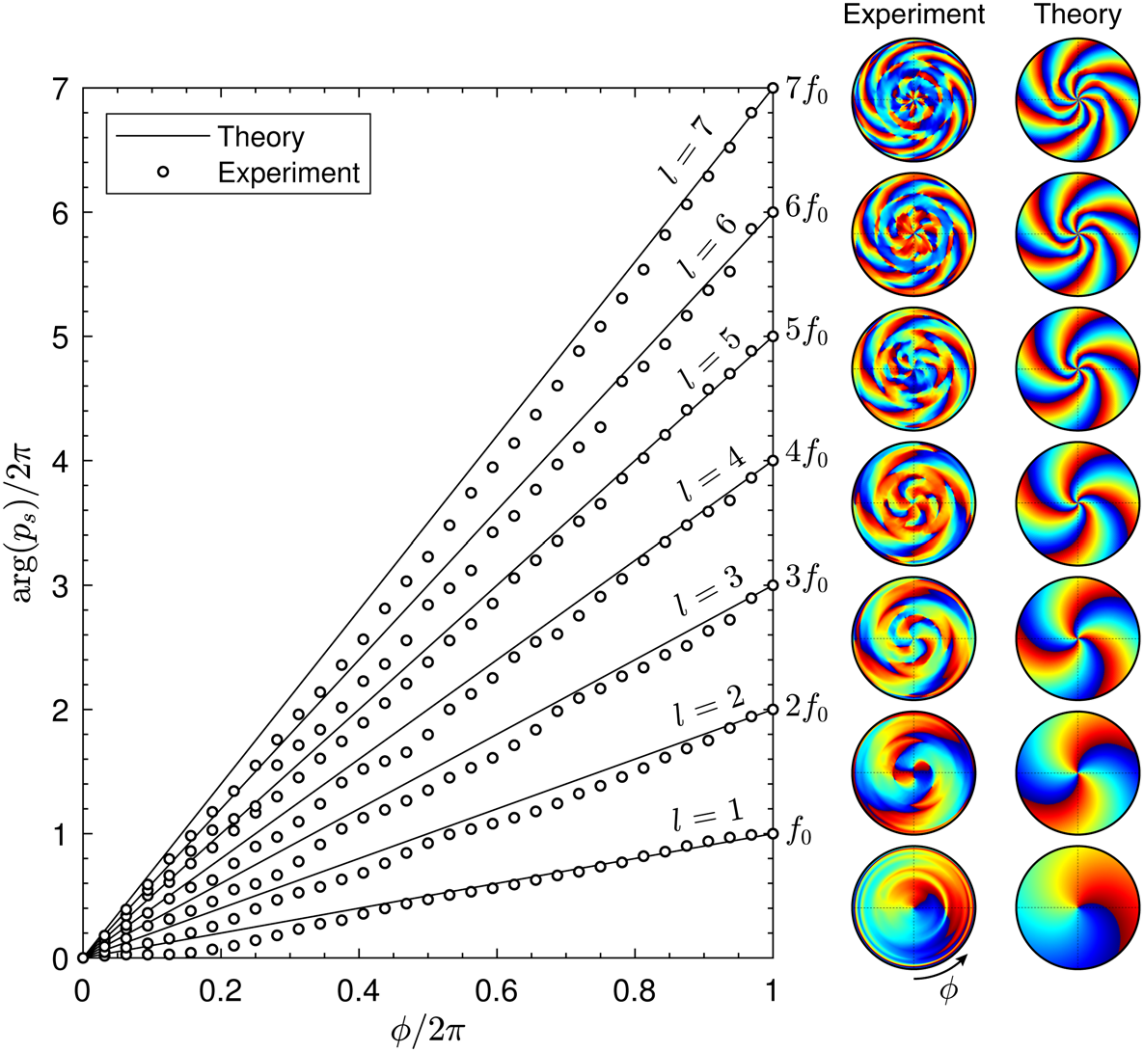
Phase of the scattered field in the far field as a function of the azimuthal angle. Each curve corresponds with a frequency and its associated topological charge of the scattered vortex, *l*, is indicated. The corresponding maps show the phase of the scattered field measured experimentally (interpolation was used here) and obtained using theory.

Finally, we show the relation between the phase of the scattered field along the azimuthal coordinate and the topological charge of the vortex. Figure 6 shows the phase measured experimentally (markers) and theoretically (lines) at different frequencies. Note phase was normalized and unwrapped. It can be observed that the scattered field by the spiral metasurface presents a phase which roughly varies linearly along the azimuthal coordinate. The slope corresponds with the topological charge, i.e., $\arg (p_s(\phi )) = f/f_0 \phi$. A detailed picture is shown in the corresponding maps at the right of Fig. 6, measured experimentally over a spherical surface of radius of 80 cm, and obtained theoretically. For low topological charges, e.g., $f/f_0<4$, the phase dislocation is clearly visible at the centre. As the width of the silent area increases with frequency (or topological charge), it becomes hard to detect the dislocation at the centre for higher frequencies. However, at grazing angles, where energy is scattered, it is visible in all maps that the phase of the scattered field rotates a number of times equal to the value of the theoretical topological charge. The process continues up to $f=f_0N/2$. At this frequency, the phase along the surface of the structure is a binary spiral of *N* arms (see Supplementary material 1). Therefore, a vortex of topological charge $l=N$ is scattered^46,49^. In our case, this was set to $f = f_0N/2 = 16$ kHz, covering the whole audible spectrum. For higher frequencies, the topological charge of the scattered vortex is given by $l = N - f/f_0$, up to $f = Nf_0$. At this frequency, the phase along the structure is constant. Therefore, the metasurface acts as a flat reflecting surface. This case is the analogous behaviour of the well-known critical frequencies of quadratic-residue diffusers^17^. In the present design, this critical frequency appears at $f = 32$ kHz, far away from the audible regime.

### Oblique incidence

**Figure 7:**
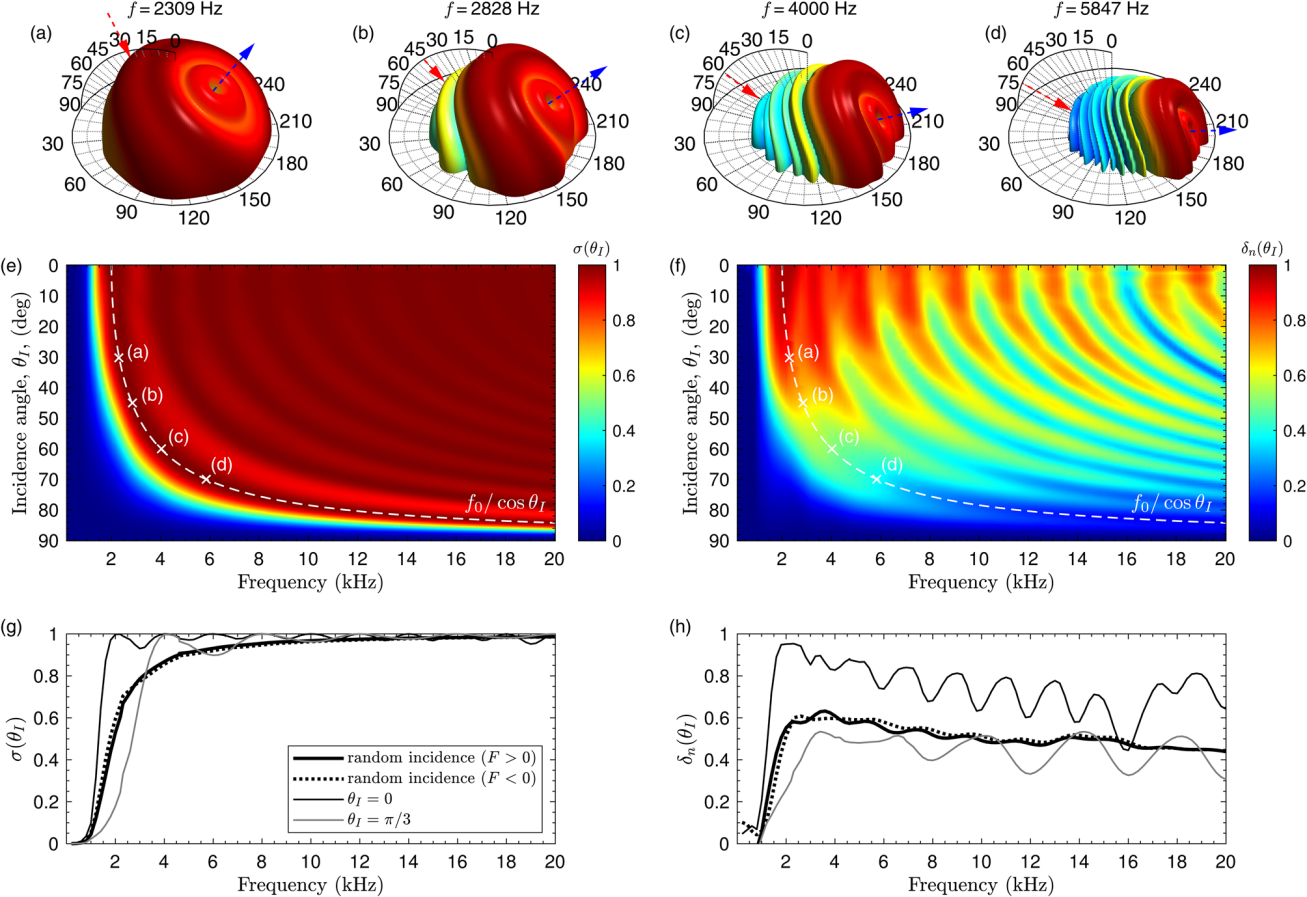
(**a1**–**d1**) Far field scattering at incidence angles $\theta _i=30^\circ , 45^\circ , 60^\circ$ and $70^\circ$ at $f=f_0$, and (**a2**–**d2**) at $f=f_0/\cos (\theta )$. (**e**) Correlation scattering coefficient and (**f**) normalized diffusion coefficient as a function of the incidence angle. (**g**) Random-incidence correlation scattering coefficient and (**h**) random-incidence normalized diffusion coefficient.

When the indent field is tilted with an oblique direction given by an angle $\theta _I$ with respect to the normal and an azimuthal angle of $\phi _I$, the scattering is being affected by two main factors. First, the transverse wavenumber is given by $k_\perp = k\sin \theta _I$, so the axial wavenumber inside each well decreases due to the conservation of the transverse component of the wavevector at the boundary, $k_z^2 = k_s^2 - k_\perp ^2$, with $k_s$ the wavenumber inside the well. Therefore, the effective wavelength increases to $\lambda _z \approx \lambda /\cos \theta _I$. As the impedance of each well is then $Z_n = -iZ_s\cot (k_z d_n)$, the quarter-wavelength resonance frequency is shifted up and the reflection coefficient along the surface becomes angle-dependent $R(x_0,y_0,\phi _I,\theta _I)$. Second, the incident field along the surface presents a sinusoidal pattern given by $p_i(x_0,y_0) = p_0\exp (-i[k_{x0}x_0 + k_{y0}y_0])$, where $k_{x0} = k_\perp \cos \phi _I$ and $k_{y0} = k_\perp \sin \phi _I$. Then, the scattered pressure at the surface is given by $p_r = p_i\,R$. Therefore, as the far-field scattering is essentially a Fourier transform of the reflected field at the surface we obtain

4 $$\begin{aligned} p_s(\phi ,\theta ,\phi _I,\theta _I) = -i\frac{k}{2\pi }\frac{\exp (ikr)}{r}\int _{S_0}p_0R(x_0,y_0,\phi _I,\theta _I) \exp (-i[(k_x-k_{x0}) x_0 + (k_y-k_{y0}) y_0])dx_0dy_0. \end{aligned}$$

Note that the product $p_i\,R$ in the spatial domain becomes a convolution in the spatial-frequency domain. This results in a shift of scattering pattern in the *k*-space by a wavevector $\mathbf{k}_\Delta = k(\cos \phi _I\sin \theta _I {\hat{k}}_x + \sin \phi _I\sin \theta _I {\hat{k}}_y)$, therefore, the scattered pattern in the far-field becomes tilted.

The two effects, the *k*-space shift and the resonance-frequency shifting, does not change the main behaviour of the metasurface. Figure 7 shows the results for oblique incidence. First, Fig. 7a1–d1 we show the scattering for $\theta _I = 30, 45, 60, 75^\circ$, at frequencies $f=f_0$, $\phi _I=0$, and for $F>0$. The far-field distribution at $f=f_0$ and low incidence angles shows a tilted and uniform pattern because wells can produce some phase shift of the reflected field. However, as the incidence angle is increased the resonance of the wells is shifted up. Therefore, at the design frequency and under very high oblique incidence, the reflection coefficient resembles the one of a rigid circular panel resulting in a poor diffusion performance.

Only when the $f=f_0/\cos (\theta _I)$ all the wells resonate according to the design and the reflection coefficient along the surface matches the holographic field of a (de)focused vortex. Therefore, as show by Fig. 7a2–d2, we recover in the far field a tilted version of the scattered vortex due to the *k*-space shift. Note in the specular direction (marked by blue arrows) the field vanish due to destructive interference. Under oblique incidence the structure also scatters vortices but at frequencies given by $f = lf_0/\cos (\theta _I)$ with topological charges given by $l=1,2,\ldots ,N/2$ for $l\le N/2$ and $l=N-1,N-2,\ldots ,0$ for $l>N/2$.

The correlation-scattering coefficient and the normalized diffusion coefficient are shown in Fig. 7e,f, respectively. First, for frequencies $f>f_0/\cos \theta _I$ one can see that the structure still show a correlation-scattering coefficient close to the unity. This is a consequence of the previous results, in this frequency range, energy is not reflected in a specular way as the structure scatters vortices. The diffusion coefficient is more affected by the incidence angle because under oblique incidence and higher frequency vortices are scattered in a narrow angular range, compare Fig. 7a2 with d2. This results in a less omni-directional response and, consequently, the diffusion coefficient decreases. To show the overall performance for oblique incidence we calculate a figure of merit, namely the random-incidence correlation-scattering and random-incidence diffusion-coefficients (see “Methods” section), where it is assumed that the probability of incidence is higher at $\theta _I=\pi /4$, as occurs in diffuse sound field. Both coefficients, shown in Fig. 7g,h, describe the performance of the structure under random incidence. Even under oblique incidence, we can see that the spiral metasurface presents a high value of both coefficients. Finally, note that the decrease in diffusing performance studied here should be a common feature of all locally-resonant metasurfaces and diffusers based on quarter-wavelength resonators.

## Conclusions

In this work, we have shown that scattered acoustic vortices present remarkable radiation properties in the far field. We have designed broadband spiral metasurfaces to generate holographic vortices. The destructive interference of the scattered waves along the axis of the spiral metasurface generates a phase dislocation with tuned topological charge in the near field. In the far field, all scattered waves at the specular direction also interfere destructively. Therefore, metasurfaces based on holographic vortices inhibit specular reflections because the field presents a phase dislocation in this direction. In addition, the scattering function in the far field is particularly uniform because the waves diverge spherically from the focal point. Moreover, under oblique incidence the present metasurface preserves the ability to scatter vortices, but at frequencies higher than the design. Therefore, the scattering patterns of spiral metasurfaces are very uniform and non-specular.

In particular, the designed metasurface presents a mean correlation-scattering coefficient of 0.99 (0.98 in the experiment) and a mean normalized diffusion coefficient of a 0.73 (0.76 in the experiment), over a frequency band covering from 2 to 20 kHz. In this way, the singular features of the resulting metasurfaces with chiral geometry allow the simultaneous generation of broadband, diffuse and non-specular scattering. These three exceptional features, as demonstrated by the outstanding values of their correlation-scattering coefficient and normalized diffusion coefficient, make spiral metasurfaces excellent candidates to generate diffuse sound reflections in practical applications of wave physics as underwater acoustics, biomedical ultrasound or room acoustics.

## Methods

### Reflection coefficient of a locally-reacting metasurface accounting for thermoviscous losses

Thermoviscous processes activate losses in the wells when they are narrow due to non-slip boundary conditions at their rigid walls. The thermal and viscous boundary layers introduce dispersion and attenuation, that are modelled using complex and frequency dependent parameters, i.e., density, $\rho (\omega )$, and bulk modulus, $K(\omega )$. For narrow slits of width $h_n=r_n-r_{n-1}-h_w$, where $h_w$ is the width of the walls between the wells, and assuming that only plane waves propagate inside them, the effective parameters are given by^51^:

5 $$\begin{aligned} \rho _n(\omega )&={\rho _0}\left[ 1-\frac{\tanh \left( \frac{h_n}{2}{G_\rho (\omega )}\right) }{\frac{h_n}{2}{G_\rho (\omega )}}\right] ^{-1} , \end{aligned}$$

6 $$\begin{aligned} K_n(\omega )&=K_0\left[ 1+(\gamma -1)\frac{\tanh \left( \frac{h_n}{2}{G_\kappa (\omega )}\right) }{\frac{h_n}{2}{G_\kappa (\omega )}}\right] ^{-1} , \end{aligned}$$

with $G_\rho (\omega )=\sqrt{{i\omega \rho _0}/{\eta }}$ and $G_K(\omega )=\sqrt{i\omega \mathrm {Pr}\rho _0/{\eta }}$, and where $\gamma$ is the ratio of the specific heats, $P_0$ is the atmospheric pressure, $\Pr$ is the Prandtl number, $\eta$ is the dynamic viscosity, and $\rho _0$ and $K_0={\gamma P_0}$ the density and bulk modulus of the surrounding and saturating fluid respectively. Considering that this fluid is the air medium, we used the parameters $\rho _0=1.213$ kg m$^{-3}$, $\mathrm {Pr}=0.71$, $\gamma =1.4$, $P_0=101325$ Pa and $\eta =1.839\;10^{-5}$ kg m$^{-1}$s$^{-1}$.

Using the complex density and bulk modulus, we can obtain the corresponding wavenumber and acoustic impedance in each well as

7 $$\begin{aligned} k_n(\omega )&= \omega \sqrt{\frac{\rho _n(\omega )}{K_n(\omega )}}, \end{aligned}$$

8 $$\begin{aligned} Z_n(\omega )&= \sqrt{\rho _n(\omega )K_n(\omega )}. \end{aligned}$$

Finally, the spatially dependent reflection coefficient of the locally reacting metasurface is given by

9 $$\begin{aligned} R(\omega ,\mathbf{r}_0) = \frac{Z_0 -i{\bar{Z}}_n\cot k_n d_n }{Z_0 + i{\bar{Z}}_n\cot k_n d_n} \end{aligned}$$

where ${\bar{Z}}_n = Z_n h_n/(h_n+h_w)$. The width, so the impedance and wavenumber, and depth of each well are calculated as a function of the position in the metasurface plane As $Z_n=Z_n(\omega ,\mathbf{r}_0)$ and $k_n=k_n(\omega ,\mathbf{r}_0)$, the reflection coefficient is spatially dependent.

### Near field calculation

The acoustic field at a point $\mathbf{r}$ scattered by the metasurface located at $\mathbf{r}_0$ at the surface $S_0$ is approximated by the Rayleigh-Sommerfeld integral and it reads as

10 $$\begin{aligned} p_s(\mathbf{r}) = -i\frac{k}{2\pi }\int _{S_0}\frac{p_0(\mathbf{r_0})R(\mathbf{r_0})\exp (ik\left| \mathbf{r} - \mathbf{r}_0\right| )}{\left| \mathbf{r} - \mathbf{r}_0\right| }dS_0, \end{aligned}$$

where $p_0(\mathbf{r}_0)$ is the incident pressure field, $R(\mathbf{r}_0)$ is the spatially-dependent reflection coefficient of the locally-reacting surface, and $k=\omega /c_0$ is the wavenumber in air at an angular frequency $\omega$, and $c_0=\sqrt{{\gamma P_0}/{\rho _0}}$ is the sound speed.

### Far field calculation

In the far field, and in spherical coordinates, $\mathbf{r}=\mathbf{r}(\phi ,\theta ,r)$, using the convention $0<\phi <2\pi$ for the azimuth and $0<\theta <\pi$ for the elevation, the distance between any point and the plane of the metasurface is approximated by

11 $$\begin{aligned} \left| \mathbf{r} - \mathbf{r}_0\right| \approx r. \end{aligned}$$

A second-order Taylor expansion gives

12 $$\begin{aligned} \left| \mathbf{r} - \mathbf{r}_0\right| \approx r - \frac{x}{r}x_0- \frac{y}{r}y_0 \approx r - \cos \phi \sin \theta x_0 - \sin \phi \sin \theta y_0. \end{aligned}$$

Introducing the approximations given by Eqs. (11) and (12) in the denominator and in the phase term of the numerator of Eq. (10), respectively, we get the Fraunhofer–Fourier approximation of the scattered field

13 $$\begin{aligned} p_s(\phi ,\theta ) = -i\frac{k}{2\pi }\frac{\exp (ikr)}{r}\int _{S_0}p_0(x_0,y_0)R(x_0,y_0) \exp (-i(k_x x_0 + k_y y_0))dx_0dy_0, \end{aligned}$$

where the transversal components of the wavevector are given by

14 $$\begin{aligned} k_x&= k \cos \phi \sin \theta ,\end{aligned}$$

15 $$\begin{aligned} k_y&= k \sin \phi \sin \theta . \end{aligned}$$

Note Eq. (13) is essentially a two-dimensional spatial Fourier transform of the reflected field and can be calculated efficiently using fast-Fourier transforms. In addition, the spherical-divergence factor ${\exp (ikr)}/{r}$ is usually dropped as it does not contribute to the directivity of the scattering in the azimuthal and elevation planes.

### Measurement procedure

**Figure 8:**
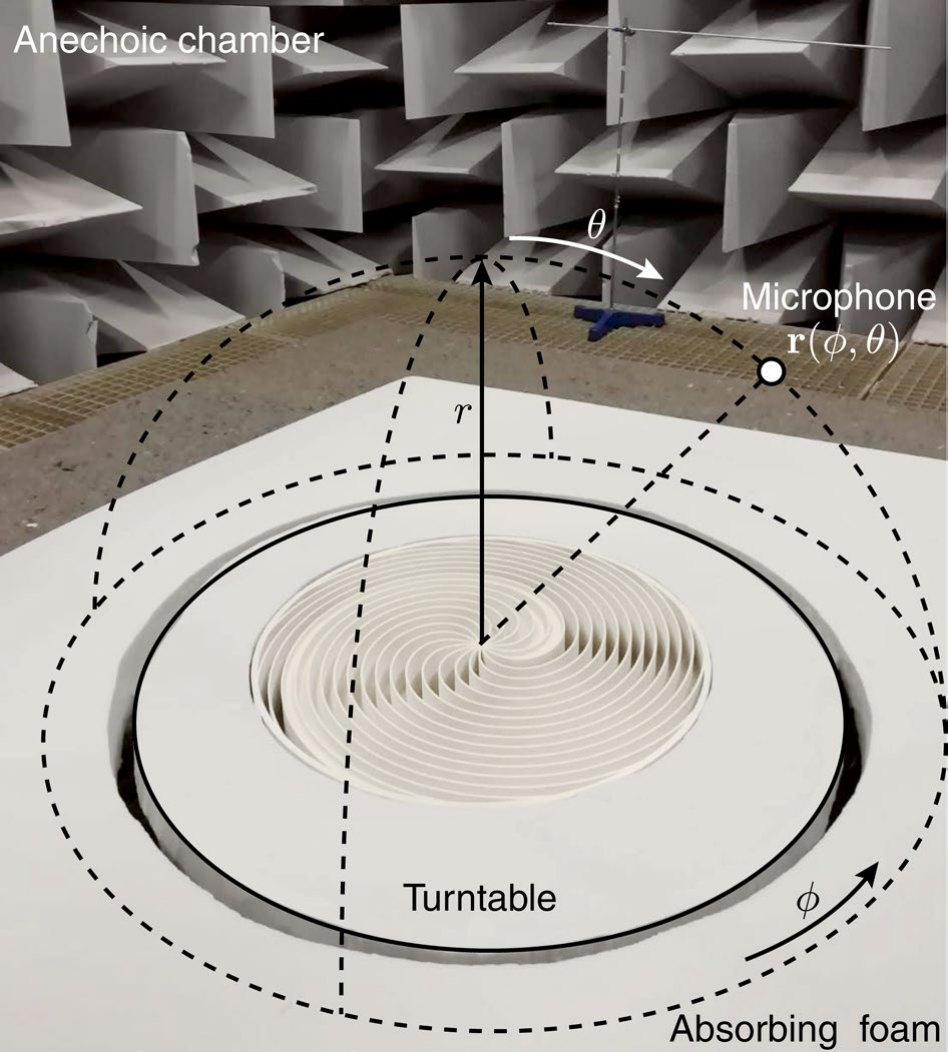
Manufactured spiral metasurface and measurement setup.

Measurements were performed in an anechoic environment following the recommendation of the standardized procedures described in Ref.^52^. Acoustic signals were acquired by a calibrated 1/4-inch pressure-field microphone (G.R.A.S. Holte, Denmark) with a preamplifier (Type 26TC, G.R.A.S. Holte, Denmark) and signal conditioning module (12AQ, G.R.A.S. Holte, Denmark). The sample was placed on a turntable (LT360, Linerx Systems). Automated measurements were performed along a uniform grid in spherical coordinates, as shown in Fig. 8, using 32 measurement points in the azimuthal direction from $0< \phi <2\pi$ ($\Delta \phi = 11.25$ deg) and 16 measurement points in the elevational direction from $0<\theta <\pi /2$ ($\Delta \theta = 6$ deg), at a distance of $r=1$ m from the center of the spiral metasurface. The turntable was surrounded by absorbing foam to avoid spurious reflections. The acoustic source was centered and located at a distance $r=2.5$ m above of the metasurface. A pseudo-random binary signal (maximum-length sequence) was used for the excitation. The system exhibited a flat response over a bandwidth ranging from 20 Hz to 20 kHz ($\pm 3$ dB).

### Sample fabrication

The spiral metasurface was 3D printed by using the sPro 230 printer (3D Systems, SC, USA). The material used for the spiral metasurface was Polyamide 12 DuraForm HST Composite (PA12 HST). The density and sound velocity of this material are respectively 1200 kg/m$^3$ and 2200 m/s. With these properties, the acoustic impedance of the material is more than 6000 times bigger than that of the air, thus it can be considered acoustically rigid. It is worth noting here that, as the far field of the focusing and defocusing metasurfaces are very similar, their diffusion is basically the same for the two cases. Thus, in this work we have 3D printed the metasurface for the focusing configuration (see Fig. 8) without any loss of generality (Fig. 7).

### Diffusion coefficient

The calculation of the diffusion coefficient follows the standardized procedures described in Ref.^52^. The acquired waveforms were deconvolved and impulse response were obtained. Temporal windowing was applied to eliminate the direct field and the spectrum at each location, $P(\omega )$, was calculated using fast-Fourier transforms. The diffusion coefficient, $\delta (\omega )$, is given by

16 $$\begin{aligned} \delta (\omega ) = \frac{\left( \sum \limits _{m=1}^M\left| P(\omega )\right| \right) ^2 - \sum \limits _{m=1}^M\left| P(\omega )\right| ^2}{\left( M-1\right) \sum \limits _{m=1}^M\left| P(\omega )\right| ^2}, \end{aligned}$$

were $M=512$ is the total number of measurements and *m* is the index of each measurement. In order to compensate the non-uniformity of the grid in spherical coordinates, the following modification^52^ is used

17 $$\begin{aligned} \delta (\omega ) = \frac{\left( \sum \limits _{m=1}^M A_m \left| P(\omega )\right| \right) ^2 - \sum \limits _{m=1}^M A_m \left| P(\omega )\right| ^2}{\left( \sum \limits _{m=1}^M A_m -1\right) \sum \limits _{m=1}^M A_m \left| P(\omega )\right| ^2}, \end{aligned}$$

where

18 $$\begin{aligned} A_m = {\left\{ \begin{array}{ll} \sin \left( \dfrac{\Delta \phi }{2}\right) , &{} \text {for } \theta _m = 0,\\ 2\sin (\theta _m)\sin \left( \dfrac{\Delta \theta }{2}\right) , &{} \text {for } 0< \theta _m < \pi /2, \\ \dfrac{4\pi }{\Delta \phi }\sin \left( \dfrac{\Delta \theta }{4}\right) ^2, &{} \text {for } \theta _m = \pi /2 . \end{array}\right. } \end{aligned}$$

The scattered field was measured for both the metasurface and a circular flat reflector, and the corresponding diffusion coefficients were calculated. Finally, the normalized diffusion coefficient, $\delta _n(\omega )$, was obtained as

19 $$\begin{aligned} \delta _n(\omega ) = \frac{\delta _s(\omega )-\delta _{r}(\omega )}{1-\delta _{r}(\omega )}, \end{aligned}$$

where $\delta _s(\omega )$ and $\delta _{r}$ are the diffusion coefficient of the spiral metasurface and the circular reflector, respectively. This coefficient measures the uniformity of the scattering.

### Correlation-scattering coefficient

Finally, the correlation-scattering coefficient $\sigma (\omega )$ was calculated using the measured scattering as^17^.

20 $$\begin{aligned} \sigma (\omega ) = 1 - \frac{\left| \sum \limits _{m=1}^M P(\omega )P_r^*(\omega )\right| ^2}{\sum \limits _{m=1}^M\left| P(\omega )\right| ^2 \sum \limits _{m=1}^M\left| P_r(\omega )\right| ^2}, \end{aligned}$$

where $P(\omega )$ and $P_r(\omega )$ are scattering of the spiral metasurface and the flat circular reflector for the *m*-th grid point, and $(^*)$ is the complex conjugate. This coefficient measures the correlation between the scattering of the structure and that of a flat panel of same dimensions.

### Random-incidence coefficients

To obtain a figure of merit under oblique incidence, we calculate the random-incidence coefficients as

21 $$\begin{aligned} \delta _{n,\text{random}}(\omega ) = \int _{\theta _I=0}^{\pi /2} \delta _n(\omega ,\theta _I) \sin (2\theta _I) d\theta _I, \quad \quad \sigma _\text{random}(\omega ) = \int _{\theta _I=0}^{\pi /2} \sigma (\omega ,\theta _I) \sin (2\theta _I) d\theta _I, \end{aligned}$$

in analogy to the random-incidence absorption coefficient^17^, where $\delta _n(\omega ,\theta _I)$ and $\sigma (\omega ,\theta _I)$ are the normalized diffusion and correlation scattering coefficients, respectively. Note in this case the reference flat circular reflector should also be calculated under oblique incidence.

## Supplementary Information

Supplementary Information

Supplementary Video 1.

Supplementary Video 2.

Supplementary Video 3.

Supplementary Video 4.

Supplementary Video 5.

Supplementary Video 6.

Supplementary Video 7.

Supplementary Video 8.
