# Supplementary material for: Spiral sound-diffusing metasurfaces based on holographic vortices
Source: Sci Rep. 2021 May 13;11:10217. doi: 10.1038/s41598-021-89487-8 (PMC8119454; doi:10.1038/s41598-021-89487-8)
Supplement: Supplementary file 1 — Supplementary Information [file 41598_2021_89487_MOESM1_ESM.pdf]

Supplementary material for: spiral sound-diffusing metasurfaces based on holographic vortices

Noé Jiménez[1,*], **Jean-Philippe Groby**[2], **and Vicent Romero-García**[2]

[1]Instituto de Instrumentación para Imagen Molecular, Universitat Politècnica de València, Consejo Superior de Invesigaciones Científicas, Camino de Vera S/N, 46085, València, Spain.
[2]Laboratoire d'Acoustique de l'Université du Mans (LAUM), UMR CNRS 6613, Institut d'Acoustique - Graduate School (IA-GS), CNRS, Le Mans Université, France.
[*]Corresponding author, nojigon@upv.es

ABSTRACT

In this document the supplementary material for the paper "spiral sound-diffusing metasurfaces based on holographic vortices" is described.

1 Scattering of vortices at non-integer multiples of the design frequency

The video scattered_vortices_by_spiral_metasurfaces_frequency_sweep.mp4 shows the far-field response of the spiral metasurface at different frequencies. The whole frequency range can be observed in the video, while some far-field responses are plotted at particular frequencies in Fig. 1 for further analysis.

It can be observed that vortices cannot be scattered by the surface if the resonance of the wells is not activated. Therefore, vortices are not generated for $f < f_0/2$. At $f = f_0/2$ the deepest well, of length $l = \lambda_0/2$, resonates at its quarter-wavelength resonance. The phase along the structure, in the near field, resembles a single spiral grating. Therefore, a vortex emerges and is visible in the far field, as it usually occurs with spiral gratings. This vortex, of single topological-charge, is visible at a grazing angle, i.e., for waves having $\sqrt{k_x^2 + k_y^2} = k$. As frequency increases, $f_0/2 < f < f_0$, the direction of the dislocation of the scattered vortex approaches the normal. At $f = f_0$ a single topological-charge dislocation is centred at the normal (see first three rows in Fig. 1).

For frequencies $f_0 < f < 2f_0$, vortex splitting-and-merging processes are observed. Eventually, phase dislocations are created at some angles and pairs of vortices of opposite topological charge emerge, see fourth row in Fig. 1. Then, near $f \lessapprox 2f_0$ two vortices of same topological charge converge to the same position in the k-space diagram. In other words, both vortices are scattered almost in the same direction. At $f = 2f_0$ the pair of vortices merge into a single topological charge and the total topological charge is the sum of the two topological charges, due to the conservation of angular momentum, in this case $l = 2$. Then, at $f \gtrapprox 2f_0$, this vortex splits again into single vortices, as shown in rows 5 to 7 in Fig. 1. This process is repeated, with increasing complexity, when frequency increases.

Note that the magnitude of the field is not strongly modified if two vortices of same topological charge are scattered in a similar direction. It is roughly equivalent to a vortex with a topological charge given by the sum of the two close vortices. This is the reason of the uniform behaviour of the spiral diffuser at non-integer frequencies. Then, around $f = 3f_0$, three single topological-charge dislocations merge into a vortex of topological charge $l = 3$.

The process continues up to $f = Nf_0/2$. At this frequency the phase along the surface of the structure is a binary spiral of N arms, therefore, a vortex of topological charge $l = N$ is scattered[44,47], see the first row of Fig. 2. In our case, this was set to $f = 16$ kHz, covering the audible spectrum. For higher frequencies the topological charge is given by $l = N - f/f_0$, up to $f = Nf_0$. At this frequency the phase along the structure is constant, therefore, the metasurface acts as a flat reflecting surface. This case is the analogous behaviour of the well-known critical frequencies of quadratic-residue diffusers, the results are shown in Fig. 2. In the present design, this frequency appears at $f = 32$ kHz, far away from the audible regime.

2 Supplementary video 1

This video (vortex_diffuser_l1.mp4) shows the time-domain representation of the scattered field by the spiral metasurface using a pulse-burst excitation of frequency $f_0 = 2$ kHz. The experimental data is compared with the theory after inverse Fourier transformation. The scattered field pattern with a vortex of integer topological charge can be identified.

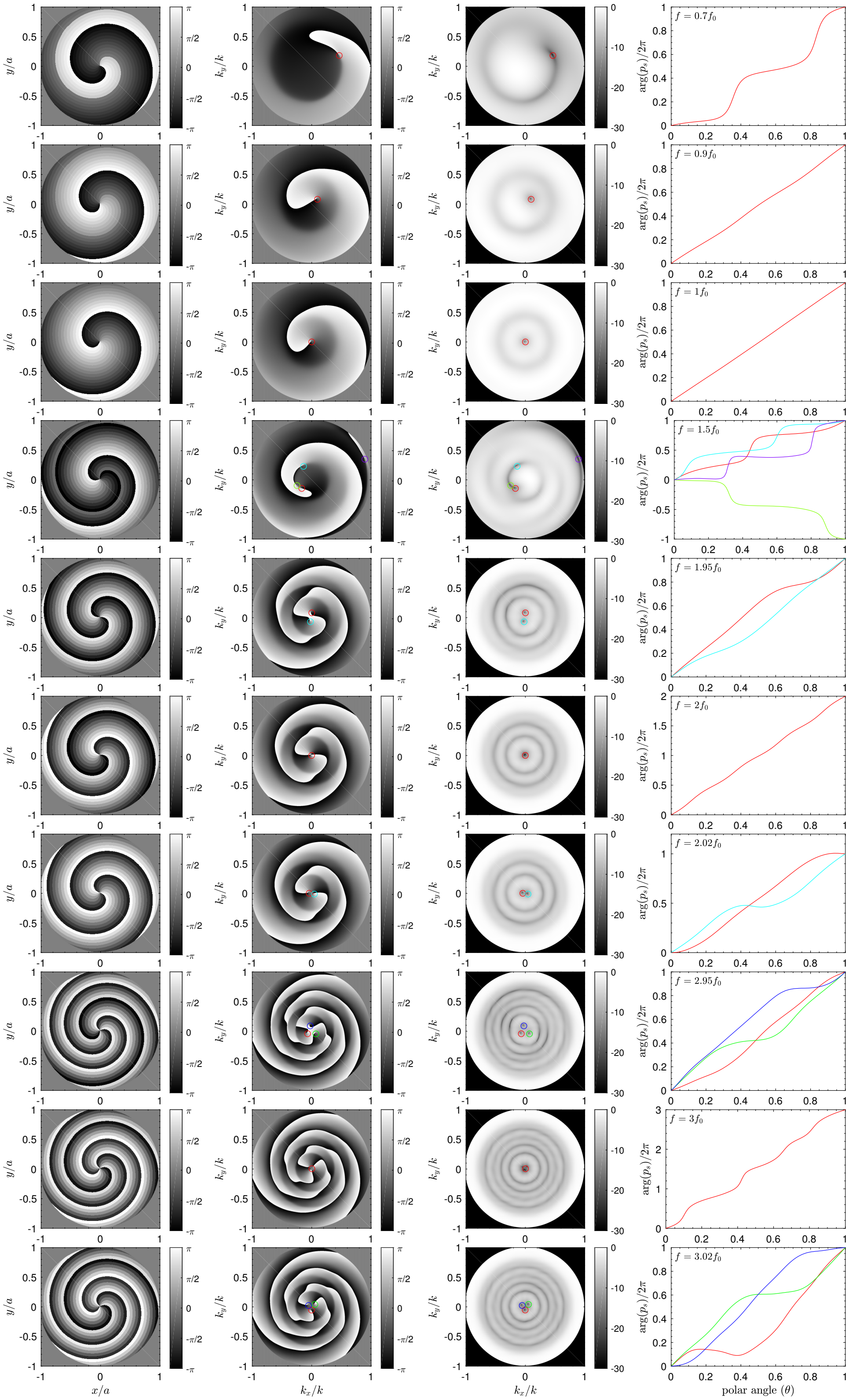

Figure 1. Scattered vortices at different frequencies. (from left to right) Phase at the surface, far-field phase, far-field magnitude, in dB, phase around the identified dislocations, marked by red circles in the far-field plots. Frequencies are indicated in the phase plot for each row.

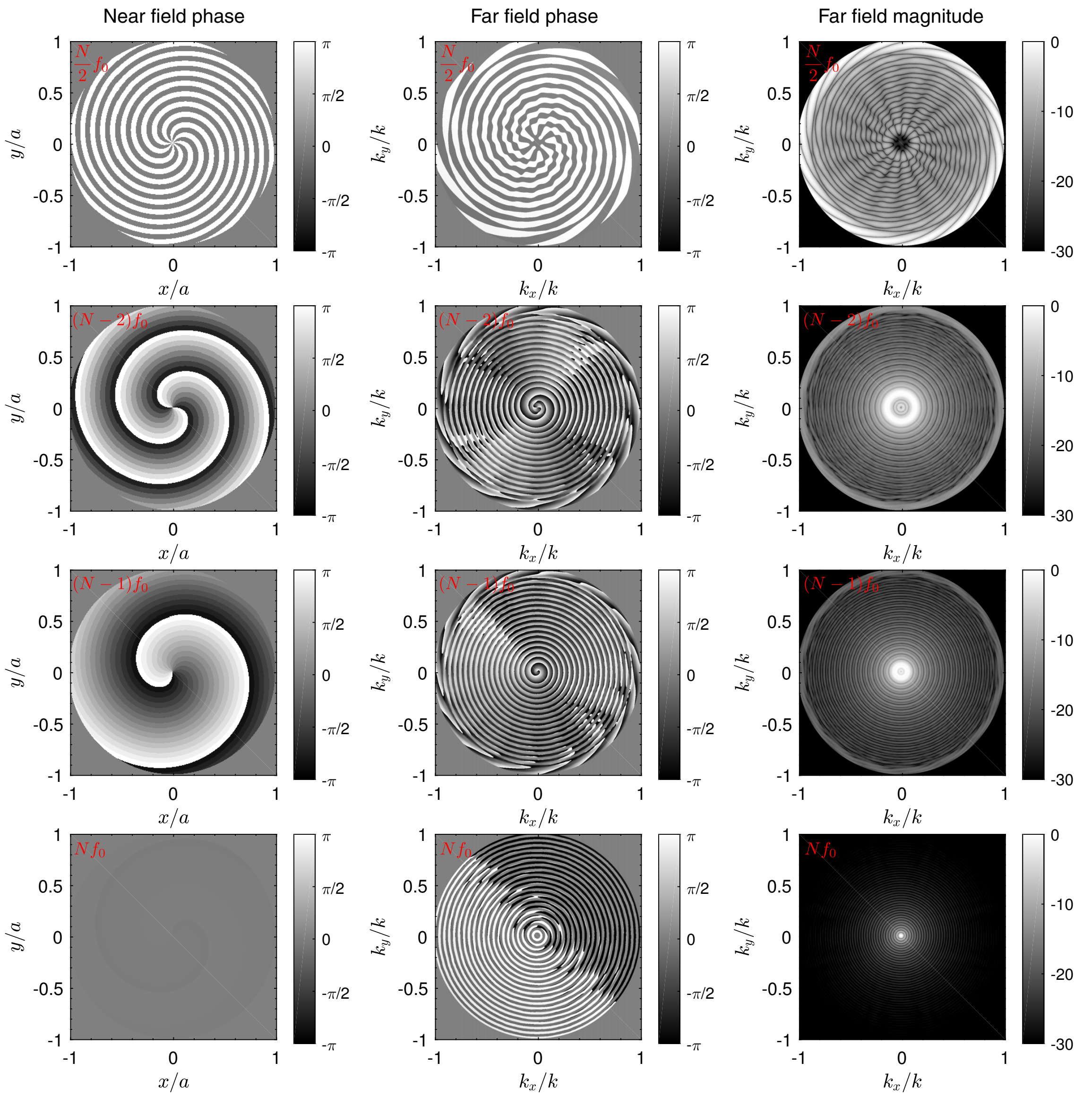

Figure 2. Scattered vortices at different frequencies. (from left to right) Phase at the surface, far-field phase, far-field magnitude, in dB. Frequencies are indicated (from top to bottom) $f = f_0 N/2, (N-2)f_0, (N-1)f_0, N f_0$.

3 Supplementary video 2

This video (vortex_diffuser_l2.mp4) shows the time-domain representation of the scattered field by the spiral metasurface using a pulse-burst excitation of frequency $f_0 = 4$ kHz. The experimental data is compared with the theory after inverse Fourier transformation. The scattered field pattern with a vortex of integer topological charge can be identified.

4 Supplementary video 3

This video (vortex_diffuser_l3.mp4) shows the time-domain representation of the scattered field by the spiral metasurface using a pulse-burst excitation of frequency $f_0 = 6$ kHz. The experimental data is compared with the theory after inverse Fourier transformation. The scattered field pattern with a vortex of integer topological charge can be identified.

5 Supplementary video 4

This video (vortex_diffuser_l4.mp4) shows the time-domain representation of the scattered field by the spiral metasurface using a pulse-burst excitation of frequency $f_0 = 8$ kHz. The experimental data is compared with the theory after inverse Fourier

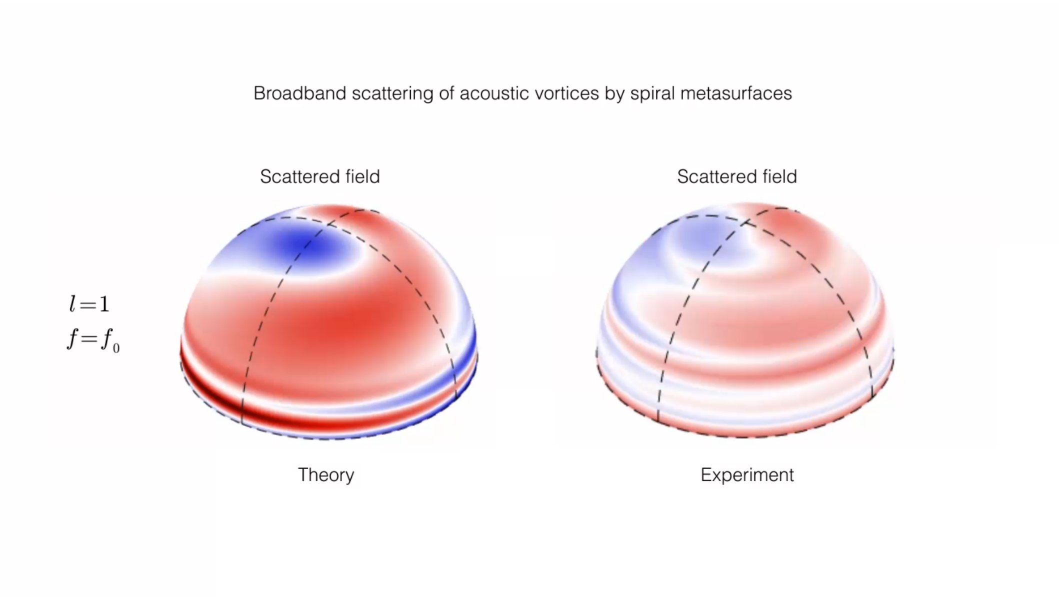

Figure 3. Frame of the supplementary video 1.

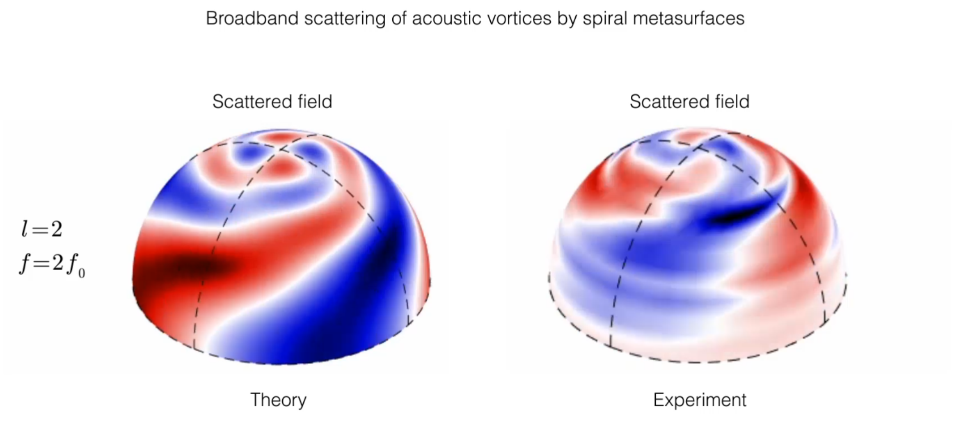

Figure 4. Frame of the supplementary video 2.

transformation. The scattered field pattern with a vortex of integer topological charge can be identified.

6 Supplementary video 5

This video (vortex_diffuser_l5.mp4) shows the time-domain representation of the scattered field by the spiral metasurface using a pulse-burst excitation of frequency $f_0 = 10$ kHz. The experimental data is compared with the theory after inverse Fourier transformation. The scattered field pattern with a vortex of integer topological charge can be identified.

7 Supplementary video 6

This video (vortex_diffuser_l6.mp4) shows the time-domain representation of the scattered field by the spiral metasurface using a pulse-burst excitation of frequency $f_0 = 12$ kHz. The experimental data is compared with the theory after inverse Fourier transformation. The scattered field pattern with a vortex of integer topological charge can be identified.

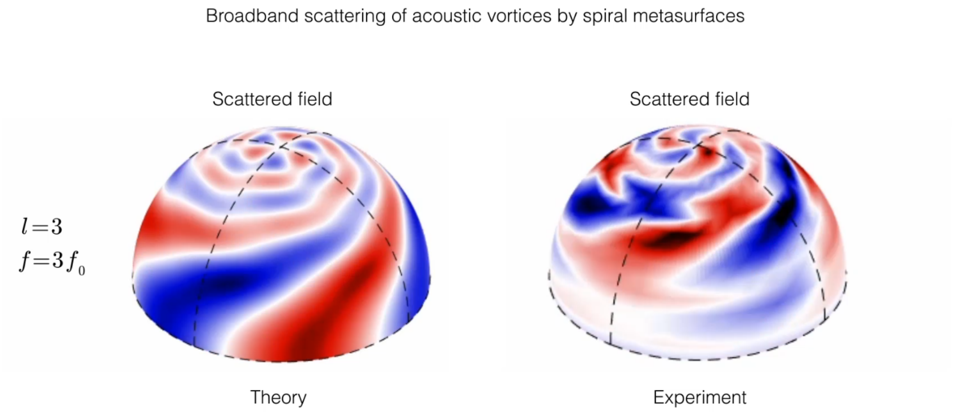

Figure 5. Frame of the supplementary video 3.

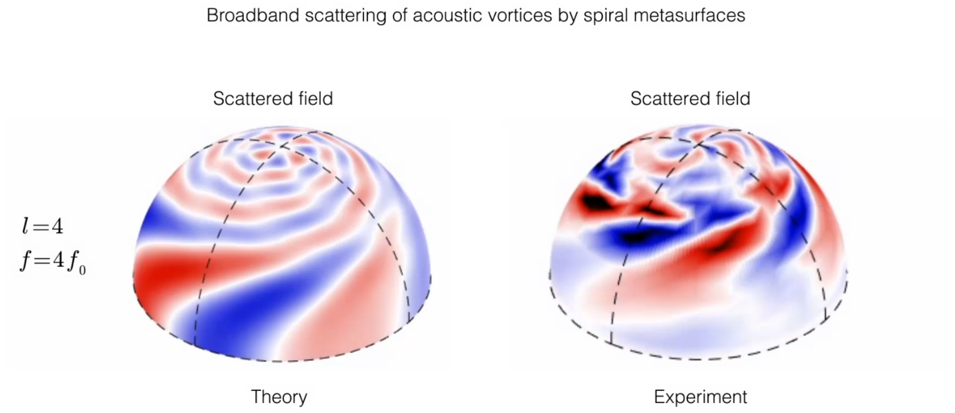

Figure 6. Frame of the supplementary video 4.

8 Supplementary video 7

This video (vortex_diffuser_l7.mp4) shows the time-domain representation of the scattered field by the spiral metasurface using a pulse-burst excitation of frequency $f_0 = 14$ kHz. The experimental data is compared with the theory after inverse Fourier transformation. The scattered field pattern with a vortex of integer topological charge can be identified.

9 Supplementary video 8

This video (vortex_diffuser_l8.mp4) shows the time-domain representation of the scattered field by the spiral metasurface using a pulse-burst excitation of frequency $f_0 = 16$ kHz. The experimental data is compared with the theory after inverse Fourier transformation. The scattered field pattern with a vortex of integer topological charge can be identified.

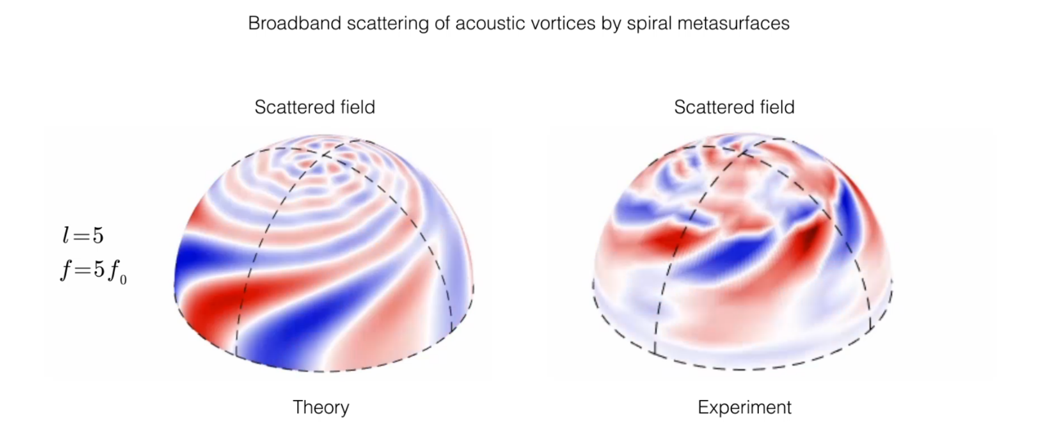

Figure 7. Frame of the supplementary video 5.

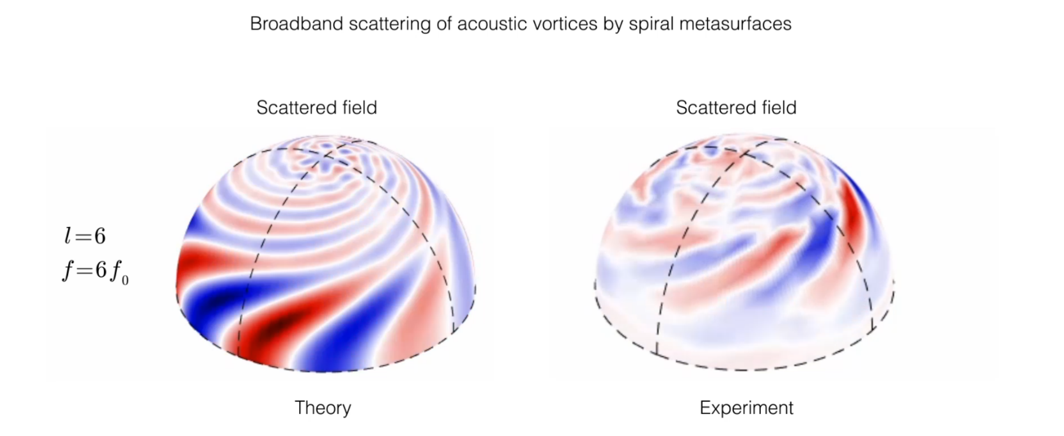

Figure 8. Frame of the supplementary video 6.

References

1. Cummer, S. A., Christensen, J. & Alù, A. Controlling sound with acoustic metamaterials. *Nature Reviews Materials* **1**, 16001 (2016).
2. Ma, G. & Sheng, P. Acoustic metamaterials: From local resonances to broad horizons. *Science advances* **2**, e1501595 (2016).
3. Assouar, B. *et al.* Acoustic metasurfaces. *Nature Reviews Materials* **3**, 460–472 (2018).
4. Zhu, Y. *et al.* Fine manipulation of sound via lossy metamaterials with independent and arbitrary reflection amplitude and phase. *Nature communications* **9**, 1–9 (2018).
5. Xie, Y. *et al.* Wavefront modulation and subwavelength diffractive acoustics with an acoustic metasurface. *Nature communications* **5**, 1–5 (2014).

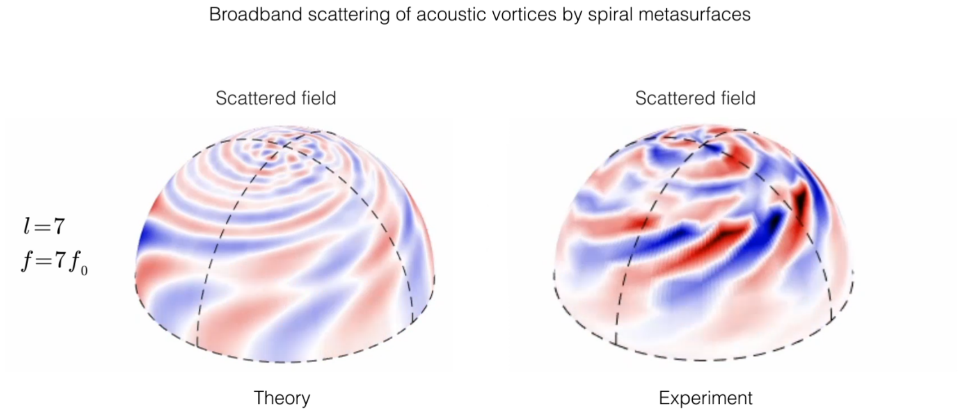

Figure 9. Frame of the supplementary video 7.

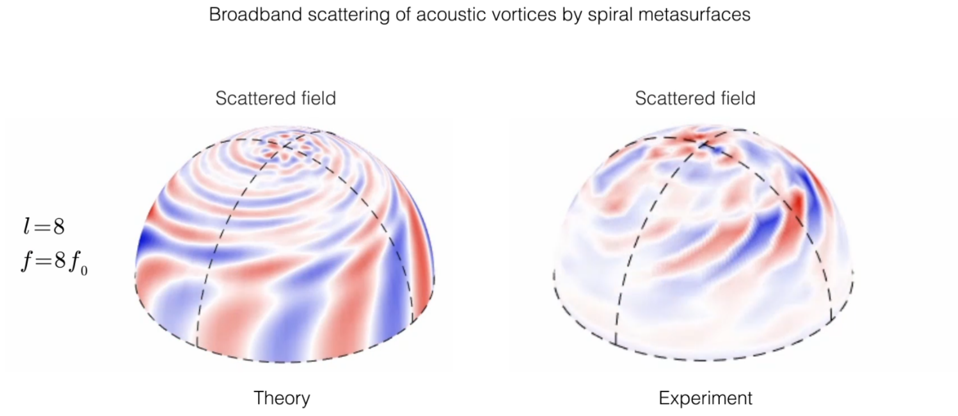

Figure 10. Frame of the supplementary video 8.

6. Li, J., Shen, C., Díaz-Rubio, A., Tretyakov, S. A. & Cummer, S. A. Systematic design and experimental demonstration of bianisotropic metasurfaces for scattering-free manipulation of acoustic wavefronts. *Nature communications* **9**, 1–9 (2018).

7. Li, Y., Liang, B., Gu, Z.-m., Zou, X.-y. & Cheng, J.-c. Reflected wavefront manipulation based on ultrathin planar acoustic metasurfaces. *Scientific reports* **3**, 2546 (2013).

8. Lemoult, F., Fink, M. & Lerosey, G. Acoustic resonators for far-field control of sound on a subwavelength scale. *Physical Review Letters* **107**, 064301 (2011).

9. Li, Y. *et al.* Experimental realization of full control of reflected waves with subwavelength acoustic metasurfaces. *Physical Review Applied* **2**, 064002 (2014).

10. Zhu, X. *et al.* Implementation of dispersion-free slow acoustic wave propagation and phase engineering with helical-structured metamaterials. *Nature communications* **7**, 1–7 (2016).

11. Zhang, S., Xia, C. & Fang, N. Broadband acoustic cloak for ultrasound waves. *Physical Review Letters* **106**, 024301 (2011).

12. Romero-García, V. *et al.* Perfect and broadband acoustic absorption by critically coupled sub-wavelength resonators. *Scientific reports* **6**, 19519 (2016).

13. Jiménez, N., Huang, W., Romero-García, V., Pagneux, V. & Groby, J.-P. Ultra-thin metamaterial for perfect and quasi-omnidirectional sound absorption. *Applied Physics Letters* **109**, 121902 (2016).

14. Jiménez, N., Romero-García, V., Pagneux, V. & Groby, J.-P. Rainbow-trapping absorbers: Broadband, perfect and asymmetric sound absorption by subwavelength panels for transmission problems. *Scientific Reports* **7**, 13595 (2017).

15. Yang, M., Chen, S., Fu, C. & Sheng, P. Optimal sound-absorbing structures. *Materials Horizons* **4**, 673–680 (2017).

16. Schröder, M. R. Diffuse sound reflection by maximum- length sequences. *The Journal of the Acoustical Society of America* **57**, 149–150 (1975).

17. Cox, T. J. & D'antonio, P. *Acoustic absorbers and diffusers: theory, design and application* (Crc Press, 2009).

18. D'antonio, P. Planar binary amplitude diffusor (1998). US Patent 5,817,992.

19. Cox, T. J., Angus, J. A. & D'Antonio, P. Ternary and quadriphase sequence diffusers. *The Journal of the Acoustical Society of America* **119**, 310–319 (2006).

20. Zhu, Y., Fan, X., Liang, B., Cheng, J. & Jing, Y. Ultrathin acoustic metasurface-based schroeder diffuser. *Physical Review X* **7**, 021034 (2017).

21. Jiménez, N., Cox, T. J., Romero-García, V. & Groby, J.-P. Metadiffusers: Deep-subwavelength sound diffusers. *Scientific reports* **7**, 5389 (2017).

22. Nye, J. & Berry, M. Dislocations in wave trains. In *Proceedings of the Royal Society of London A: Mathematical, Physical and Engineering Sciences*, vol. 336, 165–190 (The Royal Society, 1974).

23. Volke-Sepúlveda, K., Santillán, A. O. & Boullosa, R. R. Transfer of angular momentum to matter from acoustical vortices in free space. *Physical review letters* **100**, 024302 (2008).

24. Skeldon, K., Wilson, C., Edgar, M. & Padgett, M. An acoustic spanner and its associated rotational doppler shift. *New Journal of Physics* **10**, 013018 (2008).

25. Anhäuser, A., Wunenburger, R. & Brasselet, E. Acoustic rotational manipulation using orbital angular momentum transfer. *Physical review letters* **109**, 034301 (2012).

26. Demore, C. E. *et al.* Mechanical evidence of the orbital angular momentum to energy ratio of vortex beams. *Physical review letters* **108**, 194301 (2012).

27. Hong, Z., Zhang, J. & Drinkwater, B. W. Observation of orbital angular momentum transfer from bessel-shaped acoustic vortices to diphasic liquid-microparticle mixtures. *Physical review letters* **114**, 214301 (2015).

28. Wu, J. Acoustical tweezers. *The Journal of the Acoustical Society of America* **89**, 2140–2143 (1991).

29. Zhang, L. & Marston, P. L. Angular momentum flux of nonparaxial acoustic vortex beams and torques on axisymmetric objects. *Physical Review E* **84**, 065601 (2011).

30. Courtney, C. R. *et al.* Independent trapping and manipulation of microparticles using dexterous acoustic tweezers. *Applied Physics Letters* **104**, 154103 (2014).

31. Baresch, D., Thomas, J.-L. & Marchiano, R. Observation of a single-beam gradient force acoustical trap for elastic particles: acoustical tweezers. *Physical review letters* **116**, 024301 (2016).

32. Marzo, A., Caleap, M. & Drinkwater, B. W. Acoustic virtual vortices with tunable orbital angular momentum for trapping of mie particles. *Physical Review Letters* **120**, 044301 (2018).

33. Shi, C., Dubois, M., Wang, Y. & Zhang, X. High-speed acoustic communication by multiplexing orbital angular momentum. *Proceedings of the National Academy of Sciences* **114**, 7250–7253 (2017).

34. Hefner, B. T. & Marston, P. L. An acoustical helicoidal wave transducer with applications for the alignment of ultrasonic and underwater systems. *The Journal of the Acoustical Society of America* **106**, 3313–3316 (1999).

35. Thomas, J.-L. & Marchiano, R. Pseudo angular momentum and topological charge conservation for nonlinear acoustical vortices. *Physical review letters* **91**, 244302 (2003).

36. Ealo, J. L., Prieto, J. C. & Seco, F. Airborne ultrasonic vortex generation using flexible ferroelectrets. *IEEE transactions on ultrasonics, ferroelectrics, and frequency control* **58**, 1651–1657 (2011).

37. Jiang, X., Li, Y., Liang, B., Cheng, J.-c. & Zhang, L. Convert acoustic resonances to orbital angular momentum. *Physical review letters* **117**, 034301 (2016).

38. Ye, L. *et al.* Making sound vortices by metasurfaces. *AIP Advances* **6**, 085007 (2016).

39. Naify, C. J. *et al.* Generation of topologically diverse acoustic vortex beams using a compact metamaterial aperture. *Applied Physics Letters* **108**, 223503 (2016).

40. Esfahlani, H., Lissek, H. & Mosig, J. R. Generation of acoustic helical wavefronts using metasurfaces. *Physical Review B* **95**, 024312 (2017).

41. Marzo, A. *et al.* Realization of compact tractor beams using acoustic delay-lines. *Applied Physics Letters* **110**, 014102 (2017).

42. Melde, K., Mark, A. G., Qiu, T. & Fischer, P. Holograms for acoustics. *Nature* **537**, 518–522 (2016).

43. Jiménez-Gambín, S., Jiménez, N., Benlloch, J. M. & Camarena, F. Generating bessel beams with broad depth-of-field by using phase-only acoustic holograms. *Scientific Reports* **9**, 1–13 (2019).

44. Jiménez, N. *et al.* Formation of high-order acoustic bessel beams by spiral diffraction gratings. *Physical Review E* **94**, 053004 (2016).

45. Wang, T. *et al.* Particle manipulation with acoustic vortex beam induced by a brass plate with spiral shape structure. *Applied Physics Letters* **109**, 123506 (2016).

46. Jiang, X. *et al.* Broadband and stable acoustic vortex emitter with multi-arm coiling slits. *Applied Physics Letters* **108**, 203501 (2016).

47. Jiménez, N., Romero-García, V., García-Raffi, L. M., Camarena, F. & Staliunas, K. Sharp acoustic vortex focusing by fresnel-spiral zone plates. *Applied Physics Letters* **112**, 204101 (2018).

48. Stinson, M. R. The propagation of plane sound waves in narrow and wide circular tubes, and generalization to uniform tubes of arbitrary cross-sectional shape. *J. Acoust. Soc. Am.* **89**, 550–558 (1991).

49. ISO 17497-2:2012, Acoustics—Sound-Scattering Properties of Surfaces—Part 2: Measurement of the directional diffusion coefficient in a free field. International Organization for Standardization, Geneva, Switzerland, 2012.

Acknowledgements

We acknowledge financial support from the Spanish Ministry of Science, Innovation and Universities through grant “Juan de la Cierva – Incorporación” (IJC2018-037897-I) and PID2019-111436RB-C22, and by the Agència Valenciana de la Innovació through grants INNVAL10/19/016. This article is based upon work from COST Action DENORMS CA15125, supported by COST (European Cooperation in Science and Technology). JPG and VRG gratefully acknowledge the ANR-RGC METARoom (ANR-18-CE08-0021) project and the project HYPERMETA funded under the program Étoiles Montantes of the Région Pays de la Loire.

Author contributions statement

N.J. and V.R.G. conducted the theoretical modelling. N.J., J.-P.G. and V.R.G. performed the experiments. N.J., J.-P.G. and V.R.G. wrote the manuscript. All authors reviewed the manuscript.

Additional information

Supplementary information accompanies this paper at http://www.nature.com/srep
Competing interests The authors declare no competing interests.
